# Passive Exercise Adaptation for Ankle Rehabilitation Based on Learning Control Framework

**DOI:** 10.3390/s20216215

**Published:** 2020-10-31

**Authors:** Fares J. Abu-Dakka, Angel Valera, Juan A. Escalera, Mohamed Abderrahim, Alvaro Page, Vicente Mata

**Affiliations:** 1 Intelligent Robotics Group, Department of Electrical Engineering and Automation (EEA), Aalto University, 02150 Espoo, Finland; 2 Instituto Universitario de Automática e Informática Industrial (ai2), Universitat Politècnica de València, 46022 Valencia, Spain; giuprog@isa.upv.es; 3 Instituto Nacional de Técnica Aeroespacial (INTA), 28330 San Martín de la Vega, Spain; escalerapja@inta.es; 4 Department of Systems Engineering and Automation, Carlos III University of Madrid, 28911 Leganés, Spain; mohamed@ing.uc3m.es; 5 Instituto Universitario de Ingeniería Mecánica y Biomecánica, Universitat Politècnica de València, 46022 Valencia, Spain; afpage@ibv.upv.es; 6 Departamento de Ingeniería Mecánica y de Materiales, Universitat Politècnica de València, 46022 Valencia, Spain; vmata@mcm.upv.es

**Keywords:** rehabilitation robots, parallel robots, dynamic movement primitives, iterative learning control, force control, motion control

## Abstract

Ankle injuries are among the most common injuries in sport and daily life. However, for their recovery, it is important for patients to perform rehabilitation exercises. These exercises are usually done with a therapist’s guidance to help strengthen the patient’s ankle joint and restore its range of motion. However, in order to share the load with therapists so that they can offer assistance to more patients, and to provide an efficient and safe way for patients to perform ankle rehabilitation exercises, we propose a framework that integrates learning techniques with a 3-PRS parallel robot, acting together as an ankle rehabilitation device. In this paper, we propose to use passive rehabilitation exercises for dorsiflexion/plantar flexion and inversion/eversion ankle movements. The therapist is needed in the first stage to design the exercise with the patient by teaching the robot intuitively through learning from demonstration. We then propose a learning control scheme based on dynamic movement primitives and iterative learning control, which takes the designed exercise trajectory as a demonstration (an input) together with the recorded forces in order to reproduce the exercise with the patient for a number of repetitions defined by the therapist. During the execution, our approach monitors the sensed forces and adapts the trajectory by adding the necessary offsets to the original trajectory to reduce its range without modifying the original trajectory and subsequently reducing the measured forces. After a predefined number of repetitions, the algorithm restores the range gradually, until the patient is able to perform the originally designed exercise. We validate the proposed framework with both real experiments and simulation using a Simulink model of the rehabilitation parallel robot that has been developed in our lab.

## 1. Introduction

Nowadays, robots are present in many different areas. For instance, rehabilitation devices can be used as therapy aids, e.g., for the development of adjustable devices for assisting different sensorimotor functions [1,2], in schemes for improving therapeutic training [3], and for the assessment of patients’ sensorimotor performance [4]. Assistive devices have also been developed [5].

Rehabilitation robotics brings scientists from human–robot interaction and biomedical engineering together with clinicians and therapists in order to develop the necessary technologies to improve patients’ quality of life. The main goals here are [6]: (i) to develop implementable technologies that can be easily used by patients, therapists, and clinicians, (ii) to enhance the efficacy of clinicians’ therapies, and (iii) to facilitate patients’ daily activities. Moreover, using robotic systems, a very precise quantification of motion parameters can be provided by observing position, velocity, forces, etc. [7]. In order to achieve these goals, rehabilitation devices should meet some functional requirements, including: safety, stability, adaptability to the patient’s needs, accommodating a wide range of patients, providing a complete Range of Motion (ROM), being equipped with the necessary sensors for haptic and visual feedback, etc. [8,9].

In order to examine the patients’ level of adaptability while using rehabilitation devices, techniques such as passive exercise, active assisted exercise, active resistive exercise, active constrained exercise, and adaptive exercise [10] can be used, among others. Passive exercise needs no intervention by the patient and the motion is completely driven by the rehabilitation device. However, in active exercises, the patient actively interacts with the device and vice versa. Adaptive exercise refers to an excessive workout that the robot has never done, from which it tries to adapt to a new unknown pathway. During a rehabilitation treatment, cooperation between therapists and patients is required over many rehabilitation sessions in a clinic. Moreover, patients are required to continue the prescribed exercises at home. It has been documented that recovery, when using conventional treatment, is slow and sometimes takes more than a year [11]. Such a variety of rehabilitation techniques can achieve a certain level of improvement in the mobility of joints and limbs of the human body, such as the ankle joint. Some interesting examples of rehabilitation robots are MIT-MANUS for upper limb rehabilitation [11], LOKOMAT for gait training [12], and Parallel Robots (PRs) for ankle joint rehabilitation [13].

As mentioned above, the purpose of rehabilitation robotics is to design a device that mimics the work done by patient and physiotherapist during a rehabilitation session. In this paper, we propose to use a PR as a rehabilitation device. PRs have a high load capacity, stiffness, precision, compact structure, excellent energy/weight ratio, and provide better feedback control [14]. Interested readers can refer to [15,16,17,18] for more details about rehabilitation devices, modified isokinetic tables [19], gait training [20], upper limb rehabilitation [21,22], and ankle rehabilitation [23,24,25].

Regarding ankle rehabilitation systems, there are devices that generate ankle movements for neurological rehabilitation [2,26,27] or ankle sprains [13]. These devices require very precise control in order to reproduce precise movements. This control system should manage positions and forces during different exercises [28]. The Rutgers Ankle [29] was the first device used for ankle rehabilitation that provided a six-degrees-of-freedom (df) PR movement to the ankle joint. The robot applies assistive or resistive moments depending on whether the exercise is passive or active, respectively. For entertainment during exercises, the platform can be interfaced with game-like virtual environments [30]. The Rutgers Ankle is also being used to perform clinical trials for post-stroke rehabilitation [31]. Despite its use in research and experimentation, the device suffers from redundant actuations. For redundancy reduction, the authors of [13] proposed three-df and four-df PRs with a configurable central strut for sprained ankle treatments [13,32]. Different configurations of the central strut allowed the authors to analyze three different PRs in the stiffness domain.

The characteristics of the exercises to be performed in each case are very different. For that reason, a reconfigurable device is introduced in order to adapt to each patient’s range of ankle motion [33]. This robot works on the metatarsophalangeal joint and its controller varies the impedance parameters in order to accommodate different exercise modes. A three-RSS (Revolute, Spherical, and Spherical) PR is proposed by [34] and validated in simulation for ankle rehabilitation. Syrseloudis and Emiris [26] introduced a tripod-based PR actuated by electric motors for ankle rehabilitation. Actuation redundancy is used to deal with PR singularities [35]. Fan and Yin developed a four-df wearable PR [36]. In their design, the moving platform is linked to the patient’s foot, while the fixed platform is attached to the lower extremity. Cable-driven systems [37] have also been used in rehabilitation.

For more recent approaches for ankle rehabilitation using PRs, readers may refer to [38,39,40,41,42]. The selection and design of the control algorithms are based on analysis of the rehabilitation protocol taking into account the dynamics of both the system and the human–robot interaction. Dynamic posturography has been studied [43], where multi-axial perturbations are required. However, they did not use force sensor measurement.

The main motivation behind this work is to improve the therapeutic resources that can be applied to people with locomotive disorders and to offer better rehabilitation results by providing different types of exercises. In this context, it is important to develop an appropriate low-cost mechanical solution that is able to adapt different rehabilitation exercises to different patients. Unlike the aforementioned rehabilitation devices, the proposed system not only has a suitable kinematic and dynamic design but also provides a control system equipped with a learning algorithm that monitors movements and forces that arise during the execution of the exercise and that can adapt to patients’ needs using a new Learning from Demonstration (LfD) framework [44]. LfD is an end user technique for non-roboticists to teach new behaviors to a robot by extracting task-relevant information from a demonstration (or several demonstrations) and transfer these skills directly to a robot instead of hard-coding. LfD has proven to be an effective way of teaching robots important motion skills that are necessary when assisting people and providing health care services [45]. Specifically, LfD approaches have been used to teach robots a variety of skills, e.g., physical rehabilitation [46], hand rehabilitation [47], motion planning for rehabilitation [48], robotic surgery [49], and feeding [50], among others.

In this paper, we propose to exploit LfD to learn passive rehabilitation exercises and adapt them based on the patient’s needs by integrating Dynamic Movement Primitives (DMPs) [51] and Iterative learning control (ILC) [52]. DMPs are trajectory generators that can effectively encode and reproduce trajectories. DMPs were first introduced by [51], then updated in 2013 by [53], before being further updated to include unit quaternion trajectories [54]. Most recently, they have been updated to encode symmetric positive definite matrices profiles [55]. On the other hand, ILC makes it possible to reuse the control signal from the previous iteration cycle in the next one [56].

This paper is an extension of a previous conference paper [57], where we extended the theory behind the rehabilitation device and algorithm, the control scheme, stability analysis, and the validation experiments. The main contributions of this paper are:

- Exploitation of force sensing in an LfD framework for ankle rehabilitation using a PR that integrates ILC and DMPs to learn different passive exercises and adapt them autonomously;
- Implementation of soft emergency stopping due to the integration of DMP phase-stopping in the emergency button control loop in order to provide soft and smooth stopping;
- Provision of a stability analysis of our learning control;
- Provision of a brief review of ankle rehabilitation devices, injuries, and exercises;
- Implementation of different experiments in order to validate our control scheme.

## 2. Overview of the Ankle Joint: Anatomy, Physiology, and Injuries

The human foot and ankle are composed of 28 bones: tibia, fibula, 7 tarsals, 5 metatarsals, and 14 phalanges. The human ankle joint is a very complex bony structure [58] that is composed of three joints: the ankle joint proper or talocrural joint, the subtalar joint, and the inferior tibiofibular joint.

**Figure 1:**
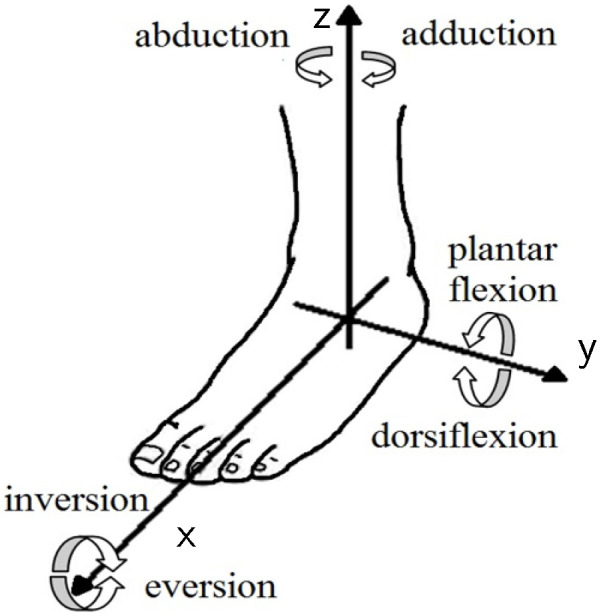
Ankle joint movements in three orthogonal planes [59].

The ankle joint has rotations in the sagittal, frontal, and transverse planes. Figure 1 shows the ankle motion in these three orthogonal planes. These motions are: (i) plantar flexion and dorsiflexion movements in the sagittal plane that occur around the *y*-axis, (ii) adduction and abduction movements in the transverse plane that occur around the *z*-axis, and (iii) inversion and eversion movements in the frontal plane that occur around the *x*-axis. The ranges and moment requirements are summarized in Table 1.

Ankle injuries are among the most common injuries in sport and daily life [58]. Ankle sprains represents 20% to 40% of all sport injuries. These sprains are a stretch or tear of the ligaments due to sudden changes in direction [64,65]. In most cases, ankle sprains can become chronic if the injury is not rehabilitated properly. Approximately 85% of ankle sprains are caused by excessive inversion [66].

The first ankle treatment after injury includes Rest, Ice, Compression, and Elevation (RICE) of the affected foot [13], which should be followed by stretching and therapy exercise along with partial weight bearing with crutches to maintain mobility in the ankle. In order to avoid muscular atrophy, which may lead to a reduction of the ROM, and stimulate healing of the injured ligaments, patient should start motion therapy within 72 h after the injury [64]. Once the ROM is achieved, strengthening of weakened muscles is essential for rapid recovery and is a preventive measure against further injury. Once patients achieve full weight-bearing capability without pain, proprioceptive exercises are initiated. These exercises aim to recover both balance and postural control using wobble boards. Finally, advanced exercises using an uneven surface wobble board should be performed to regain normal activity functions.

At present, different techniques are used for ankle rehabilitation; however, not all of them have the same effectiveness. Some techniques require the patient to be an active agent in the rehabilitation process [67]. In these cases, the patient performs active work through a series of exercises which are gradually intensified to help the ankle regain its mobility. On the other hand, the patient may also perform passive work [67,68], which usually occurs in the early stages of rehabilitation. In this type of work, an external agent, either a qualified person or a device, moves the patient’s ankle without her/his voluntary movement.

In this article, a number of references have been generated to rehabilitate an injured ankle with passive exercises. These passive exercises are used to train dorsiflexion/plantar flexion and inversion/eversion ankle movements. The rehabilitation exercises are performed using a three-PRS (Prismatic, Revolute, and Spherical) PR, which is described in the following section.

## 3. Parallel Robot: Kinematics And Dynamics

A three-df PR is used as a mechanical device for ankle rehabilitation. Its kinematic and dynamic models are explained in the following subsections.

### 3.1. Three-PRS Kinematics

**Figure 2:**
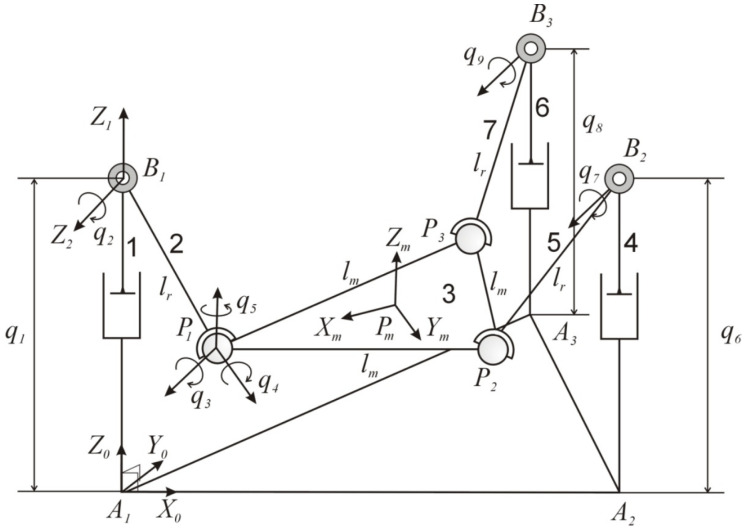
Kinematic diagram of the three-PRS, type of joints, and generalized coordinates.

The PR has been modeled by means of a set of nine dependent coordinates, as can be seen in Figure 2. These coordinates are: (i) actuated prismatic joints $\left(\underset{-}{P}\right)$ represented by $q_{1}$, $q_{6}$, and $q_{8}$, (ii) passive revolute joints (R) in $q_{2}$, $q_{7}$, and $q_{9}$, and (iii) the coordinates $q_{3}$, $q_{4}$, and $q_{5}$ only correspond to one spherical joint (S), which is located at position $P_{1}$.

Explicit expressions can be obtained for the inverse kinematic problem. Given the location of the mobile platform, so that the position and orientation of the reference system $\left\{P_{m}-X_{m}Y_{m}Z_{m}\right\}$ with respect to the fixed one $\left\{A_{1}-X_{0}Y_{0}Z_{0}\right\}$ attached to the base of the robot, it is possible to obtain the coordinates of points $P_{1}$, $P_{2}$, and $P_{3}$, corresponding to the spherical joints. These points are then used to determine the active coordinates $q_{1}$, $q_{6}$, and $q_{8}$.

The forward kinematics is solved using a geometric approach, taking into account that the length between any consecutive $P_{d}$ points is constant and equal to $l_{m}$, where d=1,2,3. Thus, the following three nonlinear geometrical constraints can be obtained:

(1) $$\begin{array}{c} \Gamma_{1}\left(q_{1,2,6,7}\right)=∥\left(r_{A_{1}B_{1}}+r_{B_{1}P_{1}}\right)-\left(r_{A_{1}A_{2}}+r_{A_{2}B_{2}}+r_{B_{2}P_{2}}\right)∥-l_{m}=0,\\ \Gamma_{2}\left(q_{1,2,8,9}\right)=∥\left(r_{A_{1}B_{1}}+r_{B_{1}P_{1}}\right)-\left(r_{A_{1}A_{3}}+r_{A_{3}B_{3}}+r_{B_{3}P_{3}}\right)∥-l_{m}=0,\\ \Gamma_{3}\left(q_{6,7,8,9}\right)=∥\left(r_{A_{1}A_{3}}+r_{A_{3}B_{3}}+r_{B_{3}P_{3}}\right)-\left(r_{A_{1}A_{2}}+r_{A_{2}B_{2}}+r_{B_{2}P_{2}}\right)∥-l_{m}=0, \end{array}$$

If we know the active generalized coordinates $q_{1}$, $q_{6}$, and $q_{8}$, it is possible to obtain the passive coordinates $q_{2}$, $q_{7}$, and $q_{9}$ by solving the equation (Equation (1)). Afterwards, the locations of points $P_{1}$, $P_{2}$, and $P_{3}$ can be easily obtained. From the coordinates of those three points, the roll γ, the pitch β angles, and the heave z of the mobile platform can be obtained.

**Figure 3:**
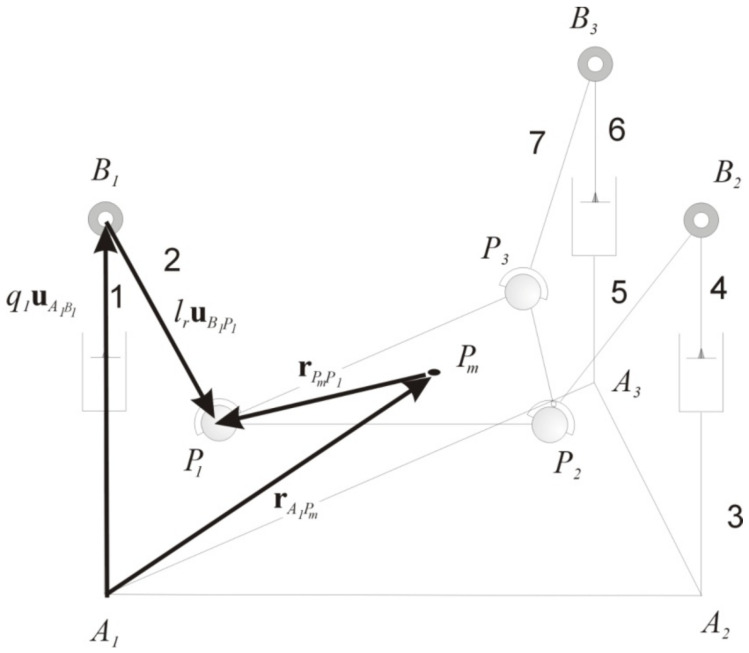
Closed loop for the velocity and acceleration problems.

The velocity problems, both inverse and forward, are based on the following:

(2) $$r_{A_{1}P_{d}}+r_{P_{d}P_{n}}=r_{A_{1}A_{n}}+q_{j}u_{A_{1},B_{n}}+l_{r}u_{B_{n},P_{n}},$$

where j=1,6, and n=1,2,3. $A_{1}$ is the fixed frame system. $P_{d}$ is the origin of the reference system attached to the mobile platform (end effector), $u_{A_{n}B_{n}}$ is a unitary vector from joints $A_{n}$ to $B_{n}$, $u_{B_{n}P_{n}}$ is a unitary vector from points $B_{n}$ to $P_{n}$, and $l_{r}$ is the constant length of links 2, 5, and 7. Figure 3 shows the closed loop for the velocity and acceleration problems for link 1; the same can be applied for links 2 and 3.

By multiplying (dot product) both sides of Equation (2) by $u_{B_{n}P_{n}}$ and taking time derivatives, the following matrix expression can be obtained. This expression relates the linear and angular velocities of the mobile platform to the time derivative of the active generalized coordinates:

(3) $$J_{x}{\left[\begin{array}{cccccc} \dot{x} & \dot{y} & \dot{z} & \omega_{x} & \omega_{y} & \omega_{z} \end{array}\right]}^{{}^{⊤}}=J_{q}\cdot {\left[\begin{array}{ccc} {\dot{q}}_{1} & {\dot{q}}_{6} & {\dot{q}}_{8} \end{array}\right]}^{{}^{⊤}}$$

where $V_{P_{m}}=\left[\dot{x}\;\dot{y}\;\dot{z}\right]{}^{⊤}$ is the velocity of the origin of the mobile reference frame (end effector). $\omega_{P_{m}}=\left[\omega_{x}\;\omega_{y}\;\omega_{z}\right]{}^{⊤}$ is the angular velocity of the mobile platform, $J_{x}$ is the Jacobian matrix in Cartesian space, and $J_{q}$ is the Jacobian matrix in generalized coordinate space.

Finally, taking into account that the parallel robot has three degrees of freedom, it is possible to obtain a relationship between the velocities of the mobile platform and the time derivatives of the roll, pitch, and heave of the reference system attached to the mobile platform and any choice of three of them forming the matrix $J_{m}$, such as:

(4) $${\left[\begin{array}{cccccc} \dot{x} & \dot{y} & \dot{z} & \dot{\phi } & \dot{\beta } & \dot{\gamma } \end{array}\right]}^{{}^{⊤}}=J_{m}\cdot {\left[\begin{array}{ccc} \dot{z} & \dot{\beta } & \dot{\gamma } \end{array}\right]}^{{}^{⊤}}$$

so that:

(5) $$J_{x}J_{m}{\left[\begin{array}{ccc} \dot{z} & \dot{\beta } & \dot{\gamma } \end{array}\right]}^{{}^{⊤}}=J_{q}\cdot {\left[\begin{array}{ccc} {\dot{q}}_{1} & {\dot{q}}_{6} & {\dot{q}}_{8} \end{array}\right]}^{{}^{⊤}}$$

This equation allows us to solve both the inverse and the forward velocity problems. In a similar way, the expressions for the acceleration can be obtained.

### 3.2. Three-PRS Dynamics

As mentioned before, the parallel robot has been modeled through a set of dependent generalized coordinates, considering which the equation of motion will be as follows:

(6) $$M\left(q,\Theta \right)\ddot{q}+C\left(q,\dot{q},\Theta \right)\dot{q}+G\left(q,\Theta \right)=\tau -J{}^{⊤}\lambda$$

where Θ is a vector grouping the dynamic parameters (masses, first inertia moments, moments and products of inertia of the links and friction coefficients). q, $\dot{q}$, and $\ddot{q}$ are the generalized coordinates, velocities, and accelerations. M stands for the mass, C for the centrifugal and Coriolis terms, while G denotes the gravitational vector. τ is the generalized torque vector. By deriving the constraint equations with respect to all generalized coordinates, we can obtain the Jacobian matrix J. λ is the vector of Lagrange multipliers. The detailed dynamic model of this PR is described in [69].

For control purposes, the generalized internal forces term is not convenient, so it could be canceled by multiplying both terms of Equation (6) by an orthogonal complement R [70]. Thus, Equation (6) can be rewritten as follows:

(7) $$R{}^{⊤}\left(M\left(q,\Theta \right)\ddot{q}+C\left(q,\dot{q},\Theta \right)\dot{q}+G\left(q,\Theta \right)\right)=R{}^{⊤}\tau$$

By considering the relationship between all the generalized coordinates and the active ones, Equation (7) can be written as follows:

(8) $$M_{df\times df}^{*}\left(q,\Theta \right){\ddot{q}}_{df\times 1}^{*}+C_{df\times df}^{*}\left(q,\dot{q},\Theta \right){\dot{q}}_{df\times 1}^{*}+G_{df\times 1}^{*}\left(q,\Theta \right)=\tau_{F\times 1}^{*}$$

where df is the number of degrees of freedom of the parallel robot, and the new vectors ${\dot{q}}^{*},{\ddot{q}}^{*}$ correspond to the active generalized velocities and accelerations.

## 4. Policy Learning and Adaptation Algorithm

In this section, we introduce our trajectory learning and adaptation algorithm for robots employed in rehabilitation activities. The proposed algorithm is general and can accommodate other robotics applications that involve contact with the environment, such as force-based trajectory tracking.

**Figure 4:**
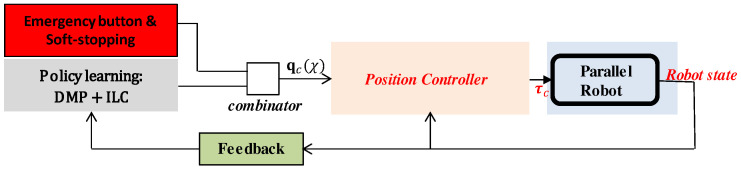
Simple diagram of the control of the proposed force-based trajectory learning and adaptation.

Figure 4 shows an illustrative diagram of the proposed framework. The position controller is fed by $q_{c}\left(\chi \right)$ (Equation (14)), which in turn results from either the emergency reference trajectory or the adapted one from the policy learning block (in gray). The policy learning block is covered in Section 4.1, Section 4.2, Section 4.3 and Section 4.4, while feedback error and offset learning is covered in Section 4.5. A more detailed diagram is shown below in Figure 6.

### 4.1. Learning from Demonstration for Rehabilitation Exercises

In this section, the patient’s ankle reference exercise trajectory is designed and the robot learning procedure of the rehabilitation trajectory is described. The trajectory is designed by a medical professional, who guides the mobile platform of the PR while the patient’s foot is in the orthopedic boot [38]. This means that the specialist moves the platform in specific directions in dorsiflexion/plantar flexion and eversion/inversion. During this movement, the trajectory of the orthopedic boot (with the patient’s foot) is measured by proprioception. The specialist moves the platform, performing an appropriate rehabilitation exercise and setting the maximum positions in each direction. These maximums are determined for each patient according to the pain that the specialist considers the patient can endure in each direction of the movement.

**Figure 5:**
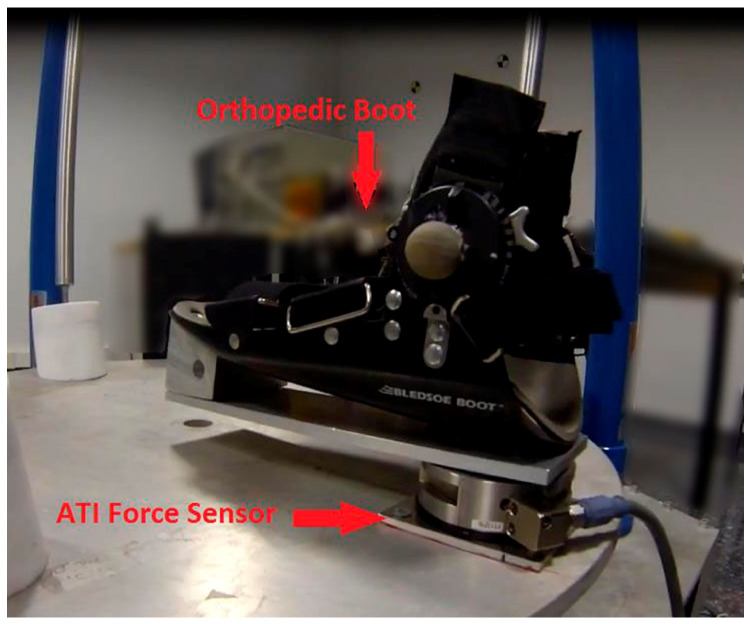
The orthopedic boot and the force sensor attached to the PR.

The force sensor is located under the orthopedic boot (Figure 5) and, consequently, the forces exerted by the specialist (human operator) during the demonstration affect the measured forces and torques. Therefore, to obtain the net forces and torques exerted by the patient, the acquired trajectory is replayed by the PR interacting solely with the patient’s foot in the boot and without any adaptation. The resulting force profiles are then recorded. These profiles indicate the actual maximum forces allowed by the patient. This procedure should be repeated for each patient.

At this stage, the exercise reference trajectory is determined. Moreover, patients should be able to repeat the exercise without any danger, because they are doing a customized exercise designed for them. In order to make the exercise more comfortable for patients and reduce the maximum forces (pain) applied to them, the specialist marks a threshold a little below those maximum forces. Thus, patients would be able to repeat the exercise assisted by the PR with less pain. After several repetitions, patients may be able to repeat the reference trajectory perfectly without pain. Afterwards, the specialist would determine whether a new exercise reference trajectory should be designed or whether the treatment should end.

### 4.2. Overview of DMPs

In this paper, robot trajectories are encoded by DMPs. They have the ability to slow execution of the trajectory (exercise) down using a phase-stopping mechanism [51] whenever it is necessary to adapt to the patient’s needs. DMPs can be found in many applications, e.g., biped locomotion [71], adaptive frequency modulation [72], reinforcement learning [73], automatic assembly [54,74,75], etc.

For each exercise, as mentioned in Section 4.1, a medical professional sets a personalized exercise reference trajectory for each patient. These trajectories are encoded by DMPs. A DMP for a single arbitrary trajectory *y* is defined by the following nonlinear differential equations [53]:

(9) $$\begin{array}{cc} \tau \dot{z} & =\alpha_{z}\left(\beta_{z}\left(y_{d}-y\right)-z\right)+f\left(\chi \right), \end{array}$$

(10) $$\begin{array}{cc} \tau \dot{y} & =z, \end{array}$$

(11) $$\begin{array}{cc} \tau \dot{\chi } & =-\alpha_{\chi }\chi , \end{array}$$

where χ is the phase variable, *z* is an auxiliary variable, and τ is the time constant. Both parameters $\alpha_{z}$ and $\beta_{z}$ define the behavior of the second-order system described by Equations (9) and (10). The phase evolution is defined by Equation (11). With the choice of the time constant τ>0, $\alpha_{z}=4\beta_{z}$, and $\alpha_{\chi }>0$, the convergence of the underlying dynamic system to a unique attractor point at $y=y_{d}$ and z=0 is guaranteed [53]. f(χ) is the linear combination of *N* nonlinear radial basis functions, which enable the robot to follow any trajectory smoothly from an initial position $y_{0}$ to a target position $y_{d}$. In the basic DMP Equations (9) and (10), each df is encoded as a separate DMP (one for dorsiflexion/plantar flexion and another for eversion/inversion); however, all the dfs share the same phase variable χ.

### 4.3. Exercise Generation Using DMPs

In order to encode exercise trajectories we substitute *y*, $y_{g}$, f(χ) in Equation (9) for q, $q_{d}$, $f_{q}\left(\chi \right)$, and $\dot{y}$ in Equation (10) for $\dot{q}$.

(12) $$f_{q}\left(\chi \right)=D\frac{\sum_{i=1}^{N}w_{i}\Psi_{i}\left(\chi \right)}{\sum_{i=1}^{N}\Psi_{i}\left(\chi \right)}\chi ,$$

where $D=\mathrm{diag}\left(q_{d}-q\right)\in R^{3\times 3}$. The diagonal matrix D is used to scale the movement amplitude if the target configuration changes. i=0,1,…,N, $\Psi_{i}\left(\chi \right)=\exp\left(-h_{i}{\left(\chi -c_{i}\right)}^{2}\right)$ are fixed basis functions. $c_{i}$ are the centers of Gaussian distributed functions throughout the phase of the trajectory and $h_{i}$ are their widths. $w_{i}$ are adjustable weights. For each df trajectory, the weights $w_{i}$ are estimated from nominal trajectories using regression [76]. Thus, the resulting DMPs encode the desired exercise trajectory. To track the desired trajectory, Equations (9) and (10) need to be integrated for all dfs with the common phase in Equation (11).

Since forces $F_{d}$ and torques $M_{d}$ (obtained from human demonstration in Section 4.1) are used as desired variables along the trajectory and not as robot control variables, they do not need to be encoded by DMPs. Instead, linear combinations of radial basis functions are used to approximate the desired forces throughout the phase $\chi_{i}=\chi \left(t_{i}\right)$:

(13) $$F_{d}\left(\chi \right)=\frac{\sum_{i}w_{i}^{F}\Psi_{i}\left(\chi \right)}{\sum_{i}\Psi_{i}\left(\chi \right)}\chi ,\;M_{d}\left(\chi \right)=\frac{\sum_{i}w_{i}^{M}\Psi_{i}\left(\chi \right)}{\sum_{i}\Psi_{i}\left(\chi \right)}\chi ,$$

Thus, six systems of linear equations need to be solved in order to estimate $w_{i}^{F}$ and $w_{i}^{M}$ from the measured force/torque data.

### 4.4. Overview of ILC

ILC [52,56] is a tracking control method for systems that execute the same trajectory in a repetitive mode. ILC assumes that the performance of an agent that repeatedly performs the same task can be improved by learning from past executions. In the conventional ILC formulation, the objective is to reduce the trajectory tacking error while rejecting periodic disturbances. This is obtained by adjusting the pre-defined control input with a corrective term that linearly depends on the tracking error.

Standard ILC assumes: (1) stable system dynamics, (2) fixed common initial conditions for each trial, and (3) the same duration for each trial. In the case of this paper, the third assumption cannot be fulfilled due to the slowing-down/speeding-up of the trajectory. However, in order to overcome this problem, the trajectory is temporarily scaled as a function of the phase variable using Equation (11). In this case, it is possible to sample the same number of times in each trial. In other word, all trials have the same phase even though they have different durations.

### 4.5. Error Feedback and DMP Phase Stopping

In human–robot interaction, the interaction might change the resulting measured forces/torques when the robot executes the demonstrated trajectory. These forces may be different from the ones (desired forces) recorded by the human demonstration. Consequently, the robot has to adapt the trajectory in order to minimize this difference between the measured and desired forces. As a solution for this problem, either admittance or impedance control can be implemented. In this work, admittance control [77] has been implemented. In human life, it can be observed that individuals are able to acquire skills in many different ways (through work, play, etc.) by repeating the same action over and over again. This means that humans learn skills from repeating actions. In the same way, and for the same task, the robot should learn from previous repetitions to adapt the executed trajectory, especially when the robot is interacting with humans for safety reasons. Hence the importance of tracking and monitoring the error in the previous repetition, which can be used to improve the performance of the next repetition for the same action (trajectory). This principle is used to learn and adapt rehabilitation exercises (trajectories) which is the basic idea of ILC [52,56].

In passive ankle rehabilitation exercises, the robot is required to follow a specific predetermined trajectory, as described in Section 4.1. However, depending on the state of the patient’s ankle, this exercise may cause some pain if the robot executes its preset trajectory. To avoid this, trajectory adaptation is introduced whenever the measured force exceeds a certain safety threshold due to the resistance of the patient’s ankle to follow the exercise. In order to adapt the PR to the new situation, the trajectory is modified according to the admittance control law [77]:

(14) $$q_{c}\left(\chi \right)=\varphi_{q}\left(\chi \right)+K\cdot e_{q}\left(\chi \right)+q_{DMP}\left(\chi \right),$$

where $q_{c}\left(\chi \right)$ is the new position commanded of the robot controller. $q_{DMP}\left(\chi \right)$ is the reference trajectory obtained by DMPs and K is the gain matrix. The force feedback control is provided by the feedback error $K\cdot e_{q}\left(\chi \right)$, where $e_{q}\left(\chi \right)=F_{d}\left(\chi \right)-F$, F and $F_{d}\left(\chi \right)$ are the actual measured force profile and the desired force one as a function of phase χ, respectively. $\varphi_{q}\left(\chi \right)$ is the on-line learned offset to be added to the original trajectory, where its initial value is $\left[0,0,0\right]{}^{⊤}$. This way, the commanded trajectory is modified by adding the offset to the original one, instead of modifying it.

To ensure safety for the patients, our proposed framework adapts the execution trajectory in order to minimize the force error between the desired and measured forces. Thus, low gains are used in order to achieve stable and robust force adaptation. Moreover, for efficient force adaptation the algorithm slow the trajectory execution down. DMP slow-down mechanism is derived from Equation (11) [51]:

(15) $$\begin{array}{cc} \tau \dot{\chi } & =-\frac{\alpha_{\chi }\chi }{1+\alpha_{p\chi }\epsilon }, \end{array}$$

(16) $$\begin{array}{cc} \tau \dot{q} & =z+\alpha_{py}\left(\tilde{q}-q\right), \end{array}$$

where $\epsilon =∥\tilde{q}-q∥$, q and $\tilde{q}$ are the DMP output and the the corresponding actual position of the robot, respectively. In this work, $\epsilon =∥e_{q}^{T}∥$, and $\alpha_{p\chi },\alpha_{py}$ are positive constants.

### 4.6. Offset Learning

The aim of our learning framework is to iteratively modify the reference trajectory so that the patient can safely repeat the rehabilitation exercise. The offset is updated after each trial through

(17) $$\delta_{t,l+1}^{q}=\varphi_{q,l}\left(\chi_{t}\right)+K\cdot e_{q}\left(\chi_{t}\right),$$

where *l* is the iterator. Each offset component $\varphi_{k}$ is represented as a linear combination of *M* radial basis functions as follows

(18) $$\varphi_{k}\left(\chi \right)=\frac{\sum_{i=1}^{M}w_{i,k}\Psi_{i}\left(\chi \right)}{\sum_{i=1}^{M}\Psi_{i}\left(\chi \right)}\chi .$$

The new data points $\{\delta_{t,l+1}^{k}\},$t=0,…,T, are obtained from the *k*-th component of the offsets trajectory, where k=1,2,3. This optimization problem aims to find $\left\{w_{i,k}\right\}$ that minimize the quadratic objective function:

(19) $$\sum_{j=0}^{T}{\left(\varphi_{k}\left(\chi_{t}\right)-\delta_{j,l+1}^{k}\right)}^{2}.$$

### 4.7. Ankle Rehabilitation Control Scheme

**Figure 6:**
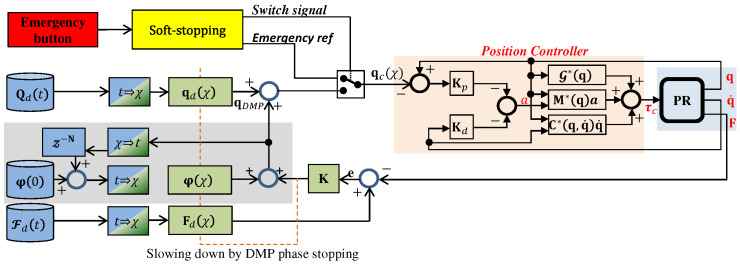
Control scheme.

A detailed control scheme of the proposed ankle rehabilitation framework is shown in Figure 6. The reference trajectory $Q_{d}\left(t\right)$ is designed by the specialist. The transformation of data from time domain to phase domain and vice versa are done in the blocks t⇒χ and χ⇒t, respectively. $q_{d}\left(\chi \right)$ is the DMP reproduction of the encoded original trajectory $Q_{d}\left(t\right)$. $F_{d}\left(t\right)$ describes the reference/desired force profile that produces $F_{d}\left(\chi \right)$ by applying Equation (13). The measured forces F may have high values depending on the patient’s response during the exercise. The objective of this work is to adapt the exercise by reducing the difference between F and $F_{d}\left(\chi \right)$. By applying Equation (14), the new offset is estimated and added to the one from the previous repetitions φ(χ).

$q_{c}\left(\chi \right)$ is the commanded trajectory to be executed by the robot and represented as the aggregation of force feedback in Equation (14), the learned offset in several repetitions through Equation (17), and the DMP-generated trajectory. This procedure is repeated until the desired and measured forces match or no further improvement is possible. The gray shaded area in Figure 6 represents the learning procedure, which belongs to the ILC algorithms, where current iteration causal learning is applied, as described in [52,56].

In our framework, we do not modify the reference/original trajectory, instead, we adapt it by adding iteratively an offset learned from the previous iteration. This offset update is represented by the discrete delay $Z^{-N}$ block in Figure 6, where N is the number of samples of that repetition.

Under normal conditions, the DMP provides the reference (heave, pitch, and roll angles) for the parallel robot control unit (see Figure 6). The controller compares the reference with the robot position/velocity to calculate the torques as in Equation (8), providing them at a frequency of 100 Hz.

It should be noted that the robot is equipped with an emergency button that can be actuated by the patient or by the specialist. If the button is pressed, the DMP stops the rehabilitation exercise and brings smoothly the platform into a safe configuration. In our case, it has been considered that the platform is at zero value for the heave as well as for the angles of eversion (roll) and dorsiflexion (pitch). When the emergency button is released, the DMP smoothly brings it to the pose where the platform was in before the button was pressed and continues with the rehabilitation exercise.

### 4.8. Stability Analysis

In order to prove the stability of the learning control, we assumed that the closed control loop of the 3-PRS is stable, without the iteration loop, with proper choice of the admittance feedback gain K [78,79]. However, closed-loop stability does not necessarily imply that the system will remain stable during the repetitive learning. In this regard, the aim of this section is to determine how our system may be affected by the iteration loop.

To clarify the notation, let uppercase letters denote one-sided *Z*-transform of the corresponding time-discrete signal, which is denoted with lower case letters. Note that the signals in Equations (14), (17) and (18) are phase dependent. However, we can always express the time-dependent counterpart and express the corresponding *Z*-transform. For the sake of simplicity, explicit dependence on *z* in transfer functions and *Z*-transform of the signals is omitted. After that, it is assumed that the nonlinear dynamics of the robot is fully compensated for using feedback control. By assuming a known environment stiffness $K_{s}$, the force at iteration *l* ($F_{l}$) can be predicted:

(20) $$F_{l}=K_{s}\left(GP_{l}-P_{o}\right),$$

where $K_{s}$ is a diagonal positive definite environment stiffness matrix, G is a diagonal matrix containing the decoupled dynamics of the robot in the form of a second-order system which maps the desired position vector $P_{l}$ into the actual position, and $P_{o}$ denotes the environment contact positions. According to Equations (14) and (17), the *Z*-transform of the error function $E_{l}$, the position update function $P_{l}$, and the learned offset function Φ are:

(21) $$\begin{array}{cc} E_{l} & =F_{d}-F_{l}, \end{array}$$

(22) $$\begin{array}{cc} P_{l} & =P_{d}+\Phi_{l}+KE_{l}, \end{array}$$

(23) $$\begin{array}{cc} \Phi_{l} & =Q\left(\Phi_{l-1}+KE_{l-1}\right). \end{array}$$

where Q presents a transfer function which maps the original sampled function to a function approximated with Gaussian kernel functions. K is the gain matrix from Equation (14). In [80] it was shown that Q can be approximated with a second-order transfer function. However, with enough *M* (in Equation (18)), the approximated function, with Gaussian kernel functions, is close enough to the original function, so that Q can be set to I. By defining $E_{l}$ as the error function and $E_{l-1}$ as the error function in the previous learning cycle [81]:

(24) $$\begin{array}{ccc} E_{l} & = & F_{d}-F_{l}\\ \; & = & F_{d}-K_{s}\left(GP_{l}-P_{o}\right)\\ \; & = & F_{d}-K_{s}\left(G\left(P_{d}+\Phi_{l}+KE_{l}\right)-P_{o}\right)\\ \; & = & F_{d}-K_{s}\left(G\left(P_{d}+\Phi_{l-1}+KE_{l-1}\right)-P_{o}\right)\\ \; & \; & -K_{s}GKE_{l}\\ \; & = & F_{d}-F_{l-1}-K_{s}GKE_{l}\\ \; & = & E_{l-1}-K_{s}GKE_{l} \end{array}$$

Dividing Equation (24) by $E_{l}$ and re-arranging leads to:

(25) $$\frac{E_{l}}{E_{l-1}}=\frac{I}{I+K_{s}GK}$$

Asymptotic stability is assured if $\frac{E_{l}}{E_{l-1}}<1,\forall \;l$. Inserting the *z* dependence into transfer functions and signals and substituting $z=e^{j\omega }$ in Equation (25), the condition for asymptotic stability becomes [52]:

(26) $$\frac{I}{I+K_{s}G\left(e^{j\omega }\right)K\left(e^{j\omega }\right)}<1;\;\forall \;\omega$$

With a proper selection of $K_{s}$ and K, the above equation is fulfilled and the learning stability is guaranteed.

## 5. Results

In this section, we develop different simulation examples as will as real experiments in order to validate the performance of our LfD framework. The trajectories used in these examples were obtained from the real robot by a medical professional guiding the moving platform of the robot, while the patient’s foot is installed in the boot, according to the procedure detailed in Section 4.1. These trajectories represent only passive exercises, which require the robot to follow them accurately.

To simulate the robot, we used a MATLAB Simulink^®^ model. The model accurately imitates the real robot [69]. The description of the robot’s hardware is detailed in the next section, where the kinematic and dynamic models used in the simulation are based on that setup.

### 5.1. Hardware Description

**Figure 7:**
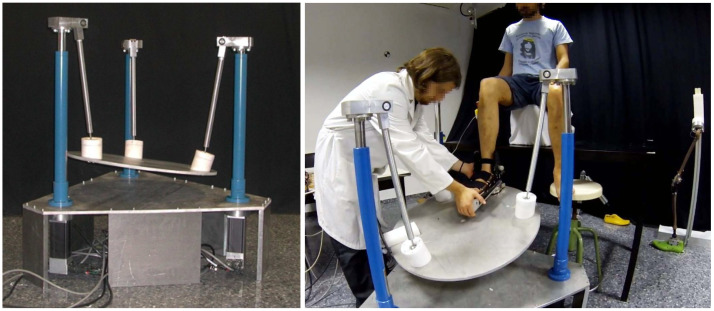
*Left*: a snapshot showing our 3-PRS robot. *Right*: a physiotherapist is fitting the patient’s foot in the orthopedic boot.

The rehabilitation robot (see Figure 7) consists of three PRS kinematic chains (1, 4, 6 in Figure 2) connected at one end to a coupling bar (2, 5, 7 in Figure 3), and at the other end they are perpendicularly attached to the platform’s base, as shown in Figure 2. Each leg is driven by a direct drive ball screw actuator (actuated prismatic joints) $\left(\underset{-}{P}\right)$. The coupling bar is connected on one side to the leg with a revolute (R) joint and with a spherical joint (S) to the moving platform on the other side. The legs are distributed forming an equilateral triangular configuration at the base. The choice of this configuration is based on the need to develop a low-cost robot with two rotational dfs that are required to perform the main rehabilitation exercises; dorsiflexion/plantar flexion and eversion/inversion. In addition, a translational df is used to adapt the platform with respect to the patient’s height while sitting on a chair. The configuration and dimensions of the robot fulfill the requirements to perform the lower limb rehabilitation exercises proposed in this paper.

The motor for each actuated prismatic joint is a brushless DC servomotor equipped with a power amplifier, with the following specifications: continuous stall torque of 2.86 Nm and continuous peak torque of 11.43 Nm. The lead of the ball screw is 20 mm and the actuators are Aerotech BMS465 AH brushless servomotors.

The control unit of the robot is based on an industrial PC equipped with two AdvantechTM cards: a PCI-1720 to supply the control actions by means of digital-to-analog outputs, and a PCI-1784, to read the actuated prismatic joint positions. The PC is equipped with a Linux Ubuntu operating system (patched with the real-time kernel Xenomai) and the open software middlewares Open RObot COntrol Software (Orocos) and Robot Operating System (ROS).

We have used open-source middlewares installed on an industrial PC for the robot control architecture. The main advantages of that are: (i) open-source with high-level tasks programming capabilities (e.g., control based on external sensing using a force sensor, automatic trajectory generation, and artificial vision, etc) and (ii) it is low-cost where the total cost of the hardware is around $2000, in addition to free operating system and programming tools.

The parallel robot is equipped with an orthopedic boot and Delta SI-330-30 ATI force sensor (Figure 5). This sensor is six-df and is capable of measuring forces and torques in 3D using a amonolithic instrumented transducer. The force/torque sensor is integrated into the system to measure the effort exerted by the patient. This configuration provides the possibility of implementing different types of rehabilitation exercises (active and passive), although this paper focuses on passive exercises. A complete description of the mechatronics of this robot can be found in [79].

### 5.2. Experiments in Simulation

#### 5.2.1. Execution of Different Exercises

**Figure 8:**
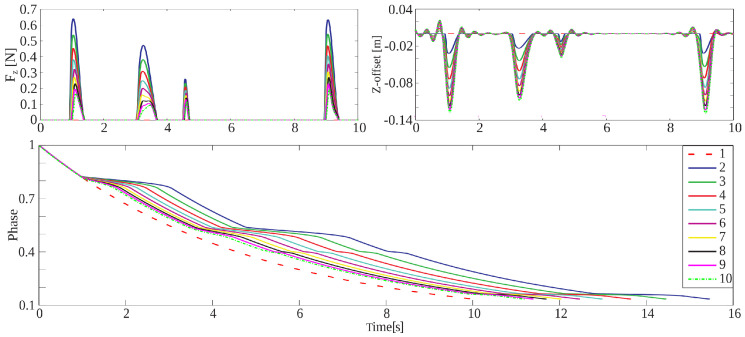
Force adaptation of the proposed framework after nine trials. *Top-right*: shows how the forces adapt, decreasing from *Line*-2 to *Line*-10. *Top-left*: represents the learned offsets, which increase throughout the trials. *Bottom*: shows the phase evolution after nine trials.

In the first simulation test, we have applied our proposed framework to learn and adapt an exercise trajectory that moves the robot platform in z while maintaining zero values for the pitch and roll angles, Figure 8. The robot moves along the nominal trajectory as long as the vertical force exerted by the patient keeps within the admissible values. Whenever the sensed forces exceeds a threshold designed by the therapist, the control system will trigger the phase-stopping mechanism in order to slow the trajectory evolution down, Equation (15). During the slow-down, a new *z*-offset will be estimated to be added to the trajectory in the next cycle which subsequently will reduce the sensed forces. Consequently, across cycles, the phase stopping becomes less frequent and the execution time decreases in each learning iteration.

If we observe Figure 8, the measured forces decrease from one trial to the next (from 2 to 10). This is because the PR adapts its trajectory whenever the measured forces exceed the desired forces. Moreover, the learned offset increases with each iteration (from 2 to 10) to adapt the trajectory to the new situation. *Line*-1 in Figure 8 corresponds to the reference force, offset, and phase. At this stage of this work, the desired force profile is equal to zero to test the functionality of the proposed algorithm on our robot.

**Figure 9:**
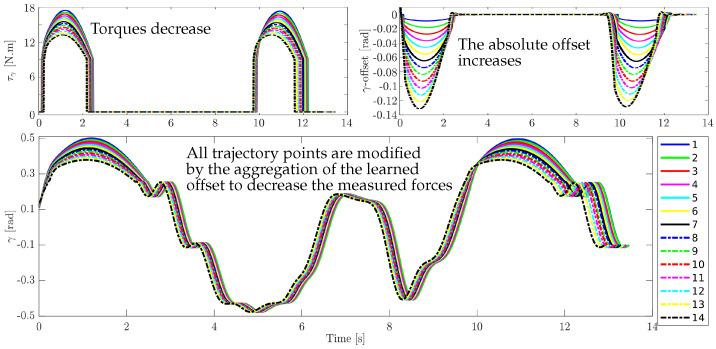
Adaptation of torques of the proposed framework after 14 trials. *Top-right*: shows torques decreasing from *Line*-1 to *Line*-14. *Top-left*: represents the learned offsets and increases throughout the trials. *Bottom*: shows adaptation of the exercise trajectory after 14 trials.

Figure 9 shows a simulation for an exercise that has been repeated 14 times. The purpose of this simulation is to show the adaptability of the system to new situations. For instance, when the measured forces are high, trajectory amplitude is reduced. The reference trajectory peaks are at 0.5 m and the corresponding forces are at 17.5 N. Now, if we set the force threshold to 13 N; which corresponds to position 0.35 m, it can be observed from the figure that the algorithm modifies the reference trajectory amplitude by adding a position offset in each repetition. This offset is calculated from the feedback forces error. After 14 repetitions, the algorithm is able to replay the trajectory into the safe region.

**Figure 10:**
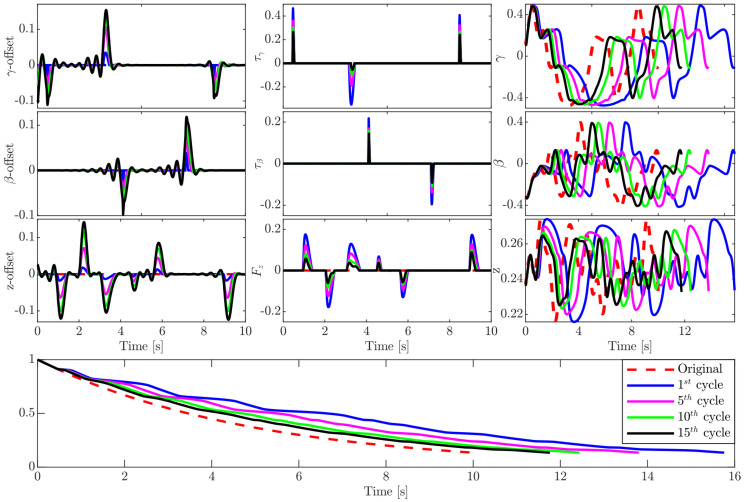
Adaptation for a three-df exercise. *Left-column*: increase of learned offset over 15 cycles. *Middle-column*: force adaptation. *Right-column*: exercise trajectory adaptation. *Bottom*: phase evolution.

The next experiment, shown in Figure 10, demonstrates a three-df exercise. In this experiment the algorithm runs γ, β, and z trajectories.

Figure 10-*bottom* shows the phase evolution of the whole exercise, the learned offset for each df is shown in Figure 10-*left*, while the force adaptation for each df is illustrated in Figure 10-*middle*. Figure 10-*right* shows the original exercise trajectory for each df as a dashed red line, the first, fifth, and fifteenth repetitions (cycles) of the same exercise. It is clear from the figure that the algorithm does not change the shape of the original trajectory. In fact, it slows the exercise down and tries to reduce the resulting forces.

It is noteworthy that when the system stabilizes and there is no further adaptation, after a period of time determined by the specialist, the algorithm starts to go backward in the direction of the original trajectory by gradually removing the added offsets. At each offset removal, the system repeats the exercise for another period of time, and so on.

#### 5.2.2. Position Tracking Error

Different model-based control strategies have been implemented for the PR, such as passivity-based control, inverse dynamic control, and adaptive control. More detailed information about the design and implementation of these controllers can be found in [79]. In addition, the authors in [78] demonstrated that the closed-loop system (robot/adaptive controller) is convergent, so the tracking error asymptotically converges to zero and all internal signals remain bounded under suitable conditions of the controller gains.

**Figure 11:**
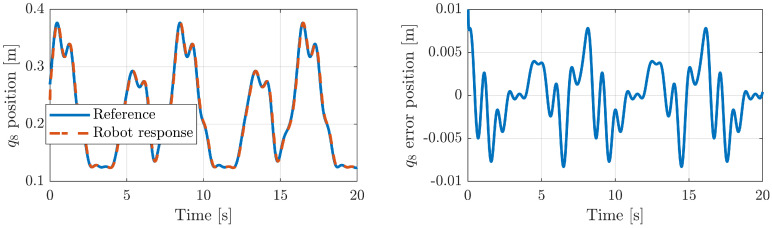
The response of the generalized coordinate $q_{8}$ in Simulink.

Figure 11 illustrates the robot response for the third active generalized coordinate $q_{8}$. Figure 11-*left* shows the reference and the robot response, while Figure 11-*right* represents the joint error. As can readily be appreciated in these figures, the robot response obtained is accurate because the robot joint follows the reference with a very small error. The other generalized coordinates, $q_{1}$ and $q_{6}$, have a very similar behavior. For verification, Table 2 shows the mean error, the root–mean–square error (RMSE) and the variance between the references and the parallel robot active joint positions.

#### 5.2.3. Emergency Button Testing

In order to validate soft-emergency-stopping, in this simulation we have used an isokinetic rehabilitation exercise based on gait trajectory training [33]. Such exercises are used to restore the original mobility and ROM and to strengthen the affected limbs or ankle joint. The ankle and foot motions are generated based on a gait of normal walking on level ground.

**Figure 12:**
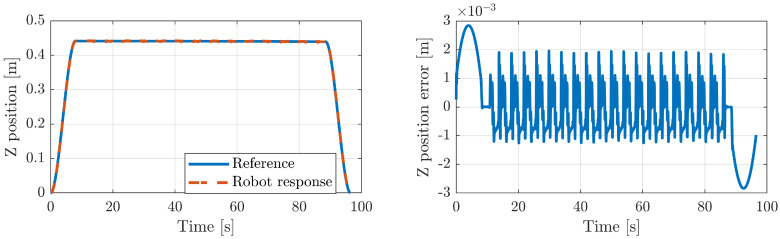
Mobile platform heave of the rehabilitation robot.

**Figure 13:**
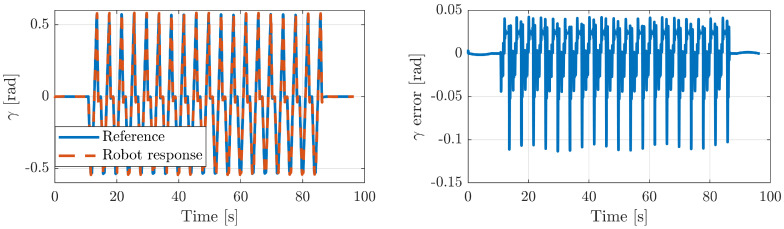
Mobile platform pitch of the Simulink model of the rehabilitation robot.

This exercise is used to restore the range of flexion/dorsiflexion motion. In order to establish the exercise, we need to elevate the platform to a certain heave. Figure 12-*left* shows the reference trajectory of the heave and the corresponding robot response, while Figure 12-*right* represents the error between both signals. In the same way, Figure 13-*left* illustrates the plantar flexion/dorsiflexion trajectory of the gait exercise along with the robot response. The error between both signals is shown in Figure 13-*left*. In all the cases, the robot controller provides very good performance, with a small error value.

**Figure 14:**
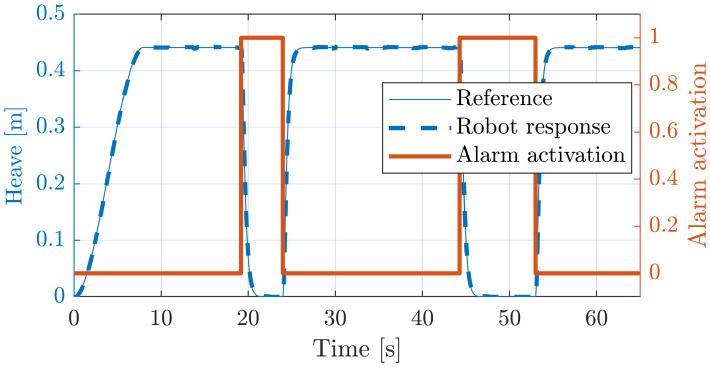
Demonstrates the effect of soft-stopping (yellow block in Figure 6) on heave evolution.

**Figure 15:**
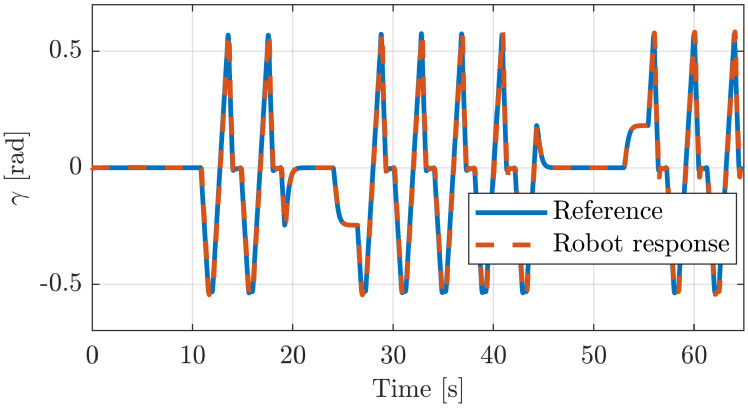
Pitch evolution with alarm activation in the Simulink model of the PR.

The following figures show another application of the DMPs with the rehabilitation robot. In this case, the patient and the medical doctor have an alarm button (red block in Figure 6). By pressing it, the control unit stops the normal execution of the exercise and moves the platform to a rest position using linear DMPs in Equation (9) with $f_{q}\left(\chi \right)=0$ (yellow block in Figure 6). As soon as the alarm button is released, the robot returns to the initial position before the alarm activation and restarts the rehabilitation exercise. The resting configuration that has been considered for the moving platform is a heave position of 0.3 m with an orientation for the roll and pitch of 0 rad. In this trial, the alarm button was pressed at time *t* = [19, 24] s. and at time *t* = [44, 53] s. Figure 14 and Figure 15 show the heave and the pitch evolution for the mobile platform in this experiment.

### 5.3. Experiments in Real Robot

**Figure 16:**
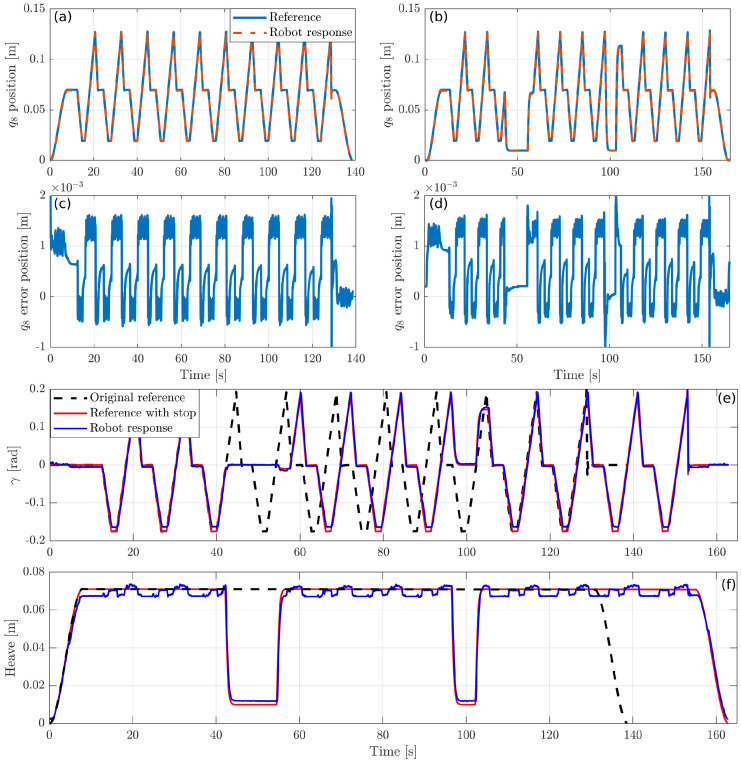
The response of the generalized coordinate $q_{8}$ of the real robot is shown in (**a**) while the effect of soft-stopping is shown in (**b**). (**c**,**d**) show the tracking error in both cases. (**e**,**f**) demonstrate the effect of soft-stopping on pitch and heave evolution.

Similarly, the previous simulated experiments (Section 5.2.3) have been tested here in real setup using the PR shown in Figure 7. Figure 16 illustrates how accurately the robot tracks a reference trajectory. Figure 16a shows the reference and the response of the active joint $q_{8}$, while (b) shows its response when the emergency button is pressed and released in two different locations during the execution.

As can be observed in Figure 16c,d, the response of the real robot is very precise, having a tracking error around 1 mm in both previous executions.

Figure 16e,f show the reference trajectory and the response of the robot for γ and the heave, respectively. In this execution, the emergency button has been activated twice, as it can be observed in the figure. This execution has been obtained with a system of 10 cameras that detect the position and orientation of the mobile platform. As we can see, the tracking for the angle γ is accurate, while the heave has a tracking error of about 3 mm due to the mechanical clearances of the robot.

## 6. Conclusions

In this paper, we proposed an LfD framework to learn and adapt passive exercises for ankle rehabilitation using a PR. This framework, exploits DMPs along with ILC in order to iteratively adapt the exercise trajectory by transferring the feedback error into an offset that can be added to the original trajectory.

Moreover, we solved the forward and inverse kinematic models for our device as well as the dynamic model and the Jacobian needed to implement force control. A model-based controller was chosen to carry out the active generalized coordinates position control. The response obtained with this position control showed an accurate response in terms of position error.

In order to validate our system, we conducted several simulation examples in addition to real experiments in order to test the adaptability, robustness, and accuracy of the system.In these tests, we used passive exercises trajectories where different movement references for γ, β, and *z* have been executed by the robot. Observing the experiments, the algorithm was able to successfully adapt the exercise to the patient’s needs by learning the offset that leads to a reduction in the measured forces exerted by the patient.

Finally, our proposed framework successfully adapts trajectories based on sensed forces. However, in the future we still need to extend this approach in two directions: (i) perform a clinical study, and (ii) speed-up the exercise execution based on the therapeutic recommendations; for example, when the patient repeats the original exercise, the algorithm starts to speed up the exercise.

## Figures and Tables

**Table 1:** ROM and moment requirement for the human ankle.

	Ankle Motion	Range of Motion ROM [60]	Maximum Passive Moment (Nm) [61,62,63]
+γ	Dorsiflexion	20.3° to 29.8°	34.1 ± 14.5
−γ	Plantarflexion	37.6° to 45.75°	48.1 ± 12.2
+β	Inversion	14.5° to 22°	33.1 ± 16.5
−β	Eversion	10,0° to 17°	40.1 ± 9.2
	Adduction	22.0° to 36°	-
	Abduction	15.4° to 25.9°	-

**Table 2:** Robot position errors (mean and RMSE) and variance.

Joint	Mean Errors	RMSE	Variance
$q_{1}$	0.00343	0.00405	1.643 × 10${}^{-3}$
$q_{6}$	0.00320	0.00396	1.567 × 10${}^{-3}$
$q_{8}$	0.00265	0.00341	1.156 × 10${}^{-3}$

## Abbreviations

The following abbreviations are used in this manuscript:

DMP	Dynamic Movement Primitive
ILC	Iterative learning control
PR	Parallel Robot
RICE	Rest, Ice, Compression, and Elevation
ROM	Range of Motion
